# Comparative Genomics of *Bordetella pertussis* Reveals Progressive Gene Loss in Finnish Strains

**DOI:** 10.1371/journal.pone.0000904

**Published:** 2007-09-19

**Authors:** Eriikka Heikkinen, Teemu Kallonen, Lilli Saarinen, Rolf Sara, Audrey J. King, Frits R. Mooi, Juhani T. Soini, Jussi Mertsola, Qiushui He

**Affiliations:** 1 Pertussis Reference Laboratory, National Public Health Institute, Turku, Finland; 2 Turku Graduate School of Biomedical Sciences, University of Turku, Turku, Finland; 3 Finnish DNA Microarray Centre, Turku Centre for Biotechnology, University of Turku, Åbo Akademi University, Turku, Finland; 4 Laboratory for Infectious Diseases and Screening, National Institute of Public Health and the Environment, Bilthoven, The Netherlands; 5 Department of Pediatrics, Turku University Hospital, Turku, Finland; Soongsil University, Republic of Korea

## Abstract

**Background:**

*Bordetella pertussis* is a Gram-negative bacterium that infects the human respiratory tract and causes pertussis or whooping cough. The disease has resurged in many countries including Finland where the whole-cell pertussis vaccine has been used for more than 50 years. Antigenic divergence has been observed between vaccine strains and clinical isolates in Finland. To better understand genome evolution in *B. pertussis* circulating in the immunized population, we developed an oligonucleotide-based microarray for comparative genomic analysis of Finnish strains isolated during the period of 50 years.

**Methodology/Principal Findings:**

The microarray consisted of 3,582 oligonucleotides (70-mer) and covered 94% of 3,816 ORFs of Tohama I, the strain of which the genome has been sequenced [1]. Twenty isolates from 1953 to 2004 were studied together with two Finnish vaccine strains and two international reference strains. The isolates were selected according to their characteristics, *e.g.* the year and place of isolation and pulsed-field gel electrophoresis profiles. Genomic DNA of the tested strains, along with reference DNA of Tohama I strain, was labelled and hybridized. The absence of genes as established with microarrays, was confirmed by PCR. Compared with the Tohama I strain, Finnish isolates lost 7 (8.6 kb) to 49 (55.3 kb) genes, clustered in one to four distinct loci. The number of lost genes increased with time, and one third of lost genes had functions related to inorganic ion transport and metabolism, or energy production and conversion. All four loci of lost genes were flanked by the insertion sequence element IS*481*.

**Conclusion/Significance:**

Our results showed that the progressive gene loss occurred in Finnish *B. pertussis* strains isolated during a period of 50 years and confirmed that *B. pertussis* is dynamic and is continuously evolving, suggesting that the bacterium may use gene loss as one strategy to adapt to highly immunized populations.

## Introduction

*Bordetella pertussis* is a Gram-negative bacterium that causes a highly contagious respiratory disease, pertussis or whooping cough. Despite extensive immunization, the disease has remained endemic causing epidemics every 3 to 5 years. A resurgence of pertussis has been observed in USA, Europe, Canada, Australia and Asia [2]–[10]. In Finland, a whole-cell pertussis vaccine was introduced in 1952. Although the vaccination coverage of four doses has been high, pertussis remains endemic and the incidence of pertussis has increased since last decade [6].

For more effective vaccination programs to prevent pertussis, it is important to understand pathogenesis and transmission of *B. pertussis*, to monitor changes in bacterial populations and to study impact of the changes on the prevention and incidence of disease. Different typing methods have been developed and applied for studying molecular epidemiology of *B. pertussis*, such as serotyping of fimbriae (Fim), genotyping of virulence genes, pulsed-field gel electrophoresis (PFGE) and multi-locus variable number of tandem repeats (MLVA) analysis [4], [6], [9], [11]–[21]. These studies revealed that the *B. pertussis* population is dynamic and that antigenic divergence has occurred with respect to pertussis toxin (Ptx), pertactin (Prn), Fim2 and Fim3 between the vaccine strains and circulating isolates. Further, it was observed that epidemics in many countries were associated with clonal expansion of strains.

Genome sequencing offers a global view of the genetic content of pathogenic bacteria. Of the nine *Bordetella* species, annotated genome sequences of *B. pertussis, B. parapertussis, B. bronchiseptica* and *B. avium* have been recently published [1], [22]. The sequenced *B. pertussis* strain Tohama I, originally isolated in Japan in 1954, is considered a reference strain and used for the production of acellular pertussis vaccines. The genome of *B. pertussis* has 3,816 ORFs [1]. A notable feature is the high load of insertion sequence (IS) elements and relatively high content (9.4%) of pseudogenes [1].

Based on the available sequence, we developed an oligonucleotide (70-mer) microarray for comparative genomic analysis of *B. pertussis* strains. Our aim was to study the evolution and molecular epidemiology of *B. pertussis* strains isolated in Finland where the whole-cell vaccine has been used for more than 50 years. Insight into *B. pertussis* capacity to adapt to vaccine-induced immunity is important for understanding the current epidemiology of pertussis in vaccinated populations. In addition to the historical perspective, the emphasis of strain selection was on recent isolates causing nationwide epidemics. The microarray results were compared with those obtained by standardized typing methods.

## Materials and Methods

### Bacterial strains and culture conditions

Bacterial isolates were selected from the *B. pertussis* strain collection from the Pertussis Reference Laboratory of the National Public Health Institute, Turku, Finland. The selection criteria included the time and geographic location of isolation, and their serotypes, genotypes and PFGE profiles [6], [13], [21]. A total of 20 clinical isolates from 1953 to 2004 together with two Finnish vaccine strains and two international reference strains, Tohama I, isolated in Japan in 1954 and 18323, isolated in USA in 1946 were included (Table 1). Finnish vaccine strain 18530 was obtained from USA and has been used since 1962, and strain 1772 was obtained from UK and was added to the vaccine in 1976. The 50-year period was covered in a way that from 1950's to 1980's one to two strains represented each decade and from 1991 onwards strains were selected according to incidence peaks observed in the whole country (Table 1). We have previously shown that Finnish *B. pertussis* population was dynamic and has continuously evolved [6], [13], [21]. Therefore, the strains selected for the present study were representative of the most prevalent PFGE profiles from 1953 to 2004. To avoid the influence of local outbreaks, isolates from at least two geographic locations were selected. All strains were isolated from children younger than 12 years.

**Table 1:** Characteristics of the bacterial strains and the distribution of absent genes detected by microarray.

Strain	Year of isolation	Sero-type	Pertactin allele	Pertussis toxin allele	PFGE-profile	Locus 4	Locus 3	Locus 1	Locus 2
						17.7 kb	22.7 kb	24.0 kb	8.6 kb
Tohama I	1954	Fim2	1	PtxA2	Tohama I	+	+	+	+
18323(1	1946		6	PtxA4	18323	+	+	+(2	+
18530(3		Fim3	1	PtxA3	BpFINR13	+	+	+	+
1772(4		Fim2,3	1	PtxA2	BpSR23	+	+	-	-
KKK22	1953	Fim3	1	PtxA2	BpFINR1	+	+	+	-
KKK1277	1964	Fim2,3	1	PtxA2	BpFINR1	+	+	+	-
KKK1330	1965	Fim2	1	PtxA2	BpFINR14	+	+	+	-
1977/3	1977	Fim3	1	PtxA1	BpSR23	+	+	-	-
1977/7	1977	Fim3	1.	PtxA1	BpFINR9	+	+	-	-
PRCB2/H	1982	Fim2	2	PtxA1	BpSR18	+	+	-	-
PRCB20/S	1982	Fim2	2	PtxA1	BpSR46	+	+	-	-
PRCB2	1991	Fim2	2	PtxA1	BpSR1	+	+	-	-
PRCB13	1992	Fim2	2	PtxA1	BpSR1	+	+	-	-
PRCB41	1993	Fim2	2	PtxA1	BpSR1	+	+	-	-
PRCB179	1996	Fim2	2	PtxA1	BpSR147	+	+	-	-
PRCB223	1996	Fim2	2	PtxA1	BpSR147	-	+	-	-
PRCB272	1999	Fim3	2	PtxA1	BpSR11	+	-	-	-
PRCB291	1999	Fim3	2	PtxA1	BpSR5	+	-	-	-
PRCB305	1999	Fim2,3	2	PtxA1	BpSR11	+	-	-	-
PRCB374	2003	Fim3	2	PtxA1	BpSR11	+	-	-	-
PRCB382	2003	Fim3	2	PtxA1	BpSR12	+	-	-	-
PRCB406	2003	Fim3	2	PtxA1	BpSR11	+	-	-	-
PRCB461	2004	Fim3	2	PtxA1	BpSR11	+	-	-	-
PRCB474	2004	Fim3	2	PtxA1	BpSR11	+	-	-	-

Range of the lost genes in loci 1–4 is BP0910A-BP0934, BP1135-BP1141, BP1948-BP1966, and BP2088-BP2103, respectively. (+) and (−) indicates presence or absence of the locus.

1. International reference strain

2. Three genes in the locus were lost (BP0910A-BP0912).

3. Strain used for the production of whole cell vaccine in Finland since 1962 and obtained from USA.

4. Strain added to the whole cell vaccine in 1976 and obtained from UK.

Bacteria stored in −70°C were recultured on Charcoal agar (Oxoid Ltd, Basingstoke, England) with 10% defibrinated sheep blood. The plates were incubated at 35–36°C for 3 days. Bacteria were harvested and suspended in distilled water. Prior to DNA isolation, the concentration of bacterial suspension was adjusted to 2×10^9^ cells/ml.

### DNA isolation, digestion and hybridization

*B. pertussis* DNA was isolated with GenElute™ Bacterial Genomic DNA Kit (Sigma-Aldrich Inc., St. Louis, USA) and digested with Sma I (10 U/µg DNA, New England Biolabs, Ipswich, USA) according to the instructions of the labeling kit BioPrime®Array CGH Genomic Labeling System (Invitrogen Life Technologies, Carlsbad, USA). The quality of digested DNA was examined by gel electrophoresis. Digested and undigested DNA was compared as the starting material for hybridization and no effect of the DNA treatment on identification of absent genes was found. The existence of restriction site (CCCGGG) of SmaI was searched into DNA sequences of the 3,582 70-mer oligonucleotides used in the microarray (see section of the oligonucleotide-based microarray). Only 55 (1.5%) oligonucleotides were found to possess the restriction site. Of them, 48 belonged to the genes identified as present, five as duplicated, and two as absent, indicating that the existence of restriction sites on oligonucleotides did not affect the identification of absent or duplicated genes in the microarray. To avoid the possible effect of length and conformation of long chromosomal DNA on labelling, the digested DNA was used for all experiments.

We next compared the amount of DNA (range: 2 to 8 µg) used as the starting material and found no effect on identification of absent genes. The amount of 4 µg was thus chosen for all experiments. The 4 µg of digested DNA of the Tohama I strain used as reference strain were labeled with Cy 3 and that of testing strain with Cy 5 (Amersham Biosciences UK Limited, Buckinghamshire, England) according to the protocol of BioPrime®Array CGH Genomic Labeling System (Invitrogen Life Technologies, Carlsbad, USA). The labeled DNA was purified with the purification kit of the same manufacturer and then combined. The concentrations of Cy3 and Cy5 were measured with ND-1000 Spectrophotometer (NanoDrop Technologies, Wilmington, USA).

The volume of labeled DNA was reduced below 15.7 µl in vacuum centrifuge, and 2.5 µl of yeast tRNA (10 mg/ml, Invitrogen Life Technologies, Carlsbad, USA), 4.3 µl of 20×SSC (1×SSC was 8.8 g/l NaCl and 4.4 g/l trisodiumcitrate) and 0.8 µl of 10% SDS were added. After denaturation at 100°C for 3 min, 1.7 µl of 10×Blocking Solution from DIG-Wash and Block Buffer set–kit (Roche, Mannheim, Germany) was added. The final volume for hybridization was adjusted with water to 25 µl. Before hybridization, slides were fixed by UV-cross linking with 90 mJ cm^−2^, washed for 1 min in 0.1% of SDS and 2 min in water to remove residual salt, and incubated at 50°C for 30 min in BSA solution containing 1% of BSA fraction V, 2×SSC and 0.1% of SDS to block nonspecific binding. The slides were further washed in 2×SSC for 3 min and then in 0.2×SSC for 3 min.

Hybridization was conducted under 22×25 mm LifterSlip ™ coverslip (Erie Scientific Company, Portsmouth, USA) in hybridization chamber at 65°C for 16 h. To control humidity inside the hybridization chamber, a strip of thin paper, humidified with approximately 100 µl of 3.4×SSC, was placed under the slide. After hybridization slides were washed at 65°C for 10 min with 1×SSC and 0.1% of SDS. The following washes were conducted at room temperature for 10 min with 0.5×SSC and 0.01% SDS, and for 3 min and 1 min with 0.1×SSC. Slides were dried in slide centrifuge and scanned with ScanArray ®5000 (PerkinElmer, Waltham, USA).

### Oligonucleotide-based microarray

The oligonucleotides used in the microarray were designed in collaboration with Operon Biotechnologies GmbH (Cologne, Germany), and an Array-Ready Oligo Set™ was purchased from the Operon. The set of 3,582 70-mer oligonucleotides, where one oligonucleotide corresponded to one gene, covered 94% of 3,816 ORFs of *B. pertussis* Tohama I [1]. Lyophilized oligonucleotides were dissolved in 50% of DMSO and 2×SSC to the concentration of 20µM. The oligonucleotides were spotted on UltraGaps™ coated slides (Corning Life Sciences, Acton, USA) with “R-ray”, an in-house spotter in the Finnish DNA Microarray Centre, the Centre for Biotechnology, Turku, Finland. Each oligonucleotide was spotted twice. A total of 20 randomized negative controls with no match to DNA of *B. pertussis* Tohama I were spotted in 8 separate spots to control non-specific binding. Contamination of printing tips and non-specific binding of DNA to slide surface was controlled by spotting water and buffer in 244 spots. Oligonucleotides representing 12 house-keeping genes of *B. pertussis* were spotted 10 times and used as positive controls to monitor the intra-array variation of the hybridization.

### Image processing, data analysis and statistical testing

**Figure 1:**
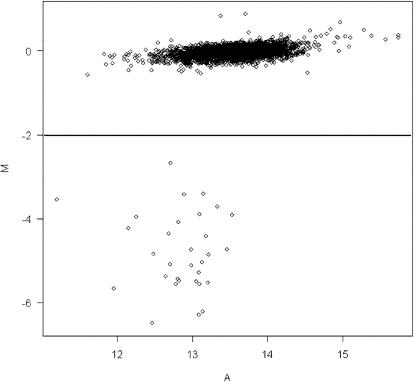
MA-plot analysis of microarray results of a *B. pertussis* clinical strain (PRCB179). The mean of intensity (A) is plotted against log-transformed intensity ratio (M) of individual spots. Two distinct clusters of the genes are shown. The genes present in both reference and testing strains were centered on zero, and the genes lost in the testing strain formed a separate cluster defined by M-value<−2.

Image processing was made using ScanAlyze program from Michael B. Eisen, (Univerity of California at Berkeley). MA-plot, where mean of intensity (A) is plotted against log-transformed intensity ratio (M) of each spot, was created for classifying the genes present or absent in the testing strain. In the MA-plot, majority of the M-values was centered to zero, and a clearly separated group of genes was formed with low M-values (Figure 1). As the group was completely separated from the majority, genes included in the group were considered as absent and filtered from the raw data by defining M-value<−2. Student's t-test was used to determine the difference in log-ratios between present and absent genes and p-value<0.05 considered the statistically significant.

### Confirmation of microarray results with PCR

PCR was used to confirm absent genes of the testing strains identified by microarray. The primers were designed with Primer3 program (Steve Rozen&Helen J. Skaletsky, 2000) on the basis of the two genes which flanked the lost locus or of the two genes located at each end of the lost locus. The sequences of the primers and their target genes were shown in Table S1.

PCR reaction contained 5–50 ng of purified DNA, 200 µM of each dNTP (Promega, Madison, WI, USA), 20 pmol of primers (Sigma Aldrich, Haverhill, UK), 5% DMSO (Merck, Darmstadt, Germany), 1×Buffer II, 1.5–3 mM of MgCl_2_, 0.625–2.5 U of AmpliTaq Gold® DNA polymerase (Applied Biosystems, Foster City, USA). The reaction volume was adjusted to 50 µl with Molecular Biology grade water (Eppendorf, Hamburg, Germany). PCR was run in MJ Research PTC 200 Thermal Cycler (Global Medical Instrumentation, Ramsey, USA) with the following protocol: initial denaturation at 95°C for 8 min followed by 30–38 cycles of 30 sec at 94°C, 30 sec to 1 min at 60–62°C and 1 to 3 min at 72°C, and with a final elongation of 5 min at 72°C. The expected size of PCR products was confirmed by gel electrophoresis.

## Results

### Population of *B. pertussis* in Finland

**Figure 2:**
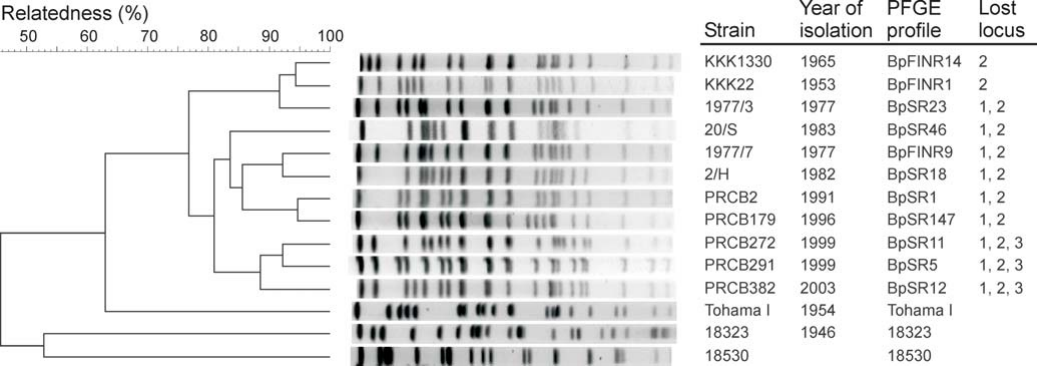
Dendrogram analysis of PFGE profiles of *B. pertussis* clinical and reference strains. The DNA profiles obtained after restriction with XbaI are shown, and only different PFGE profiles identified among the strains tested by microarray were included. The unweighted pair group method using arithmetic averages (UPGMA) with 1% band tolerance and 1% optimization settings was used as the clustering method.

More than 400 clinical isolates collected since 1953 have been analyzed by serotyping of Fim, genotyping of pertussis toxin S1 subunit (*ptxA*) and pertactin (*prn*), and PFGE [6], [13], [21]. The strains isolated in 1953–1965 had the same *ptxA* as the vaccine strains (*ptxA2*), but a change to *ptxA1* occurred in the 1970's (Table 1). During the 1980's, a change from *prn1* (the vaccine type) to *prn2* (non-vaccine type) was observed. We have previously shown that Finnish *B. pertussis* population was dynamic and has continuously evolved [6], [21]. The most prevalent PFGE profiles found during the period of 50 years were BpFinR1 in 1953–1965, BpSR23 in 1970s and BpSR18 in 1980's (Figure 2). From 1991 to 2004 the predominant profiles were BpSR1, BpSR147 and BpSR11 (Figure 2, Table 1). BpSR11 strains started to emerge in 1999, and its frequency reached 55% in 2003 and 42% in 2004, when the nationwide epidemic of pertussis occurred (unpublished data). Along with BpSR11, BpSR12 emerged in 2000, and the highest frequency (16%) for the profile was detected in 2003. Another profile that co-emerged with BpSR11 was BpSR5, for which the frequency in 1999 was 7%. PFGE profiles of all clinical isolates analyzed were clearly different from that of Tohama I strain, strain 18323 and the Finnish vaccine strain 18530 (Figure 2) [6], [21]. The other Finnish vaccine strain 1772 represented BpSR23, the predominant PFGE profile in 1970s. The relatedness between Tohama I and strain 18323 was less than 50% (Figure 2).

### Performance of the microarray

The oligonucleotide-based microarray was proved to be specific for *B. pertussis* because no hybridization was observed to randomized negative control spots or water spots. Only marginal variation in the log-ratios was observed when self to self hybridization of DNA of the reference strain and dye swap of DNA of the testing strain were tested.

The 12 housekeeping genes with multiple spots (ten per array) were used for the determination of intra-array variation. The mean of signal intensity ratio (reference strain *vs* testing strain) ranged from 1.03 to 1.29 and SD from 0.04 to 0.08. The coefficient of variation (CV) was thus 3.37 to 6.76%. Number of absent genes detected was proved to be constant among all the replicate spots. For the determination of inter-array variation, 12 virulence genes present in the testing strain were chosen. Six individual results from three separate arrays were calculated. The mean of signal intensity ratio (reference strain *vs* testing strain) ranged from 0.99 to 1.40 and SD from 0.04 to 0.09. The CV was thus 3.52 to 8.38%.

### Gene loss in *B. pertussis* strains

Compared with the strain Tohama I, four distinct loci were identified to be absent among the Finnish clinical isolates (Table 1). Relative to the Tohama I chromosome, the number of genes absent was 24 (size: 24 kb) for locus 1 (range: BP0910A-BP0934), seven (8.6 kb) for locus 2 (BP1135-BP1141), 18 (22.7 kb) for locus 3 (BP1948-BP1966), and 16 (17.7 kb) for locus 4 (BP2088-BP2103). However, the four loci were present in the international reference strain 18323 and in the Finnish strain 18530 that has been used for vaccine production since 1962. The other strain, 1772 that was added to the vaccine in 1976, missed the loci 1 and 2. Microarray data has been deposited in Gene Expression Omnibus database (http://www.ncbi.nlm.nih.gov/projects/geo/) and is accessible through accession number GSE8092.

Twenty clinical isolates from the period 1953 to 2004 were analyzed with the microarrays. All three strains analyzed from 1953–1965 missed only one locus (Locus 2). All nine strains analyzed from 1977 to 1996 missed two loci (Loci 1 and 2). Finally, all eight strains analyzed from 1999 to 2004 missed the Loci 1 and 2 in addition to Locus 3. Altogether, the eight strains had 44 absent genes (55.3 kb) in the three different loci. Only the strain PRCB223 which was isolated in 1996 was found to miss Locus 4 (Table 1). The absence of the four lost loci was confirmed by PCR (Table S2).

Based on genome sequence of the strain Tohama I, all the four absent loci were flanked by IS*481*.

Although the four absent loci identified among the 20 clinical isolates were present in the reference strain 18323 and the Finnish vaccine strain 18530, additional absent loci were found in the two strains (Dataset S1). Strain 18323 had 196 absent genes identified in 23 loci and the number of genes in each locus ranged from 1 to 33. Of the 23 absent loci, nine were bordered by IS*481* and 14 had IS*481* adjacent to one side. The Finnish vaccine strain 18530 had 22 absent genes in five different loci and the number of genes in each locus ranged from 1 to 9 (Dataset S1). Of the five loci, four were bordered by IS*481* and one had IS*481* adjacent to one side. Two loci (BP2627-BP2629 and BP3104-BP3110) were absent in both strains 18323 and 18530. A single gene (BP1225), flanked on one side by IS*481*, was absent in two clinical strains KKK1277 and 1330, isolated in 1964 and 1965, respectively.

### Gene duplication in *B. pertussis* strain

Of the 20 clinical isolates, one strain KKK1330 was found to have both gene loss and duplication. This strain was isolated in 1965 and had a PFGE profile BpFINR14 distinct from the prevalent profile BpFinR1 found in 1950s and 1960s (Figure 2, Table 1). The strain missed locus 2 (BP1135-BP1141), whereas two loci were duplicated (i.e. BP1288-BP1442 and BP1481-BP1487). The mean of log-transformed intensity (mean±SD: 0.48±0.22) of all the duplicated genes was significantly higher than that of all other present genes (−0.36±0.26, p<0.001). When the mean of log-transformed intensity was compared between the duplicated genes and all other present genes of the strain KKK1277 isolated in 1964, no significant difference was observed (p = 0.181). IS*481* was found adjacent to one side of the locus BP1288-BP1442.

### Characteristics of the absent and duplicated genes

In the genome of the strain Tohama I, the percentage of the pseudogenes was 9.4% and average of the GC contents was 67.6% [1]. Of the 65 lost genes identified in 4 different loci, 11 (16.9%) were pseudogenes. The average percentage of pseudogenes is significantly higher than that in the genome of Tohama I strain (chi-squared test, p = 0.047). The corresponding percentages in the loci were 28.6%, 16.7%, 30.8% and 12.5%, respectively. The GC content of the lost genes in each locus was 69.6%, 66.0%, 70.4% and 68.9%, respectively.

**Figure 3:**
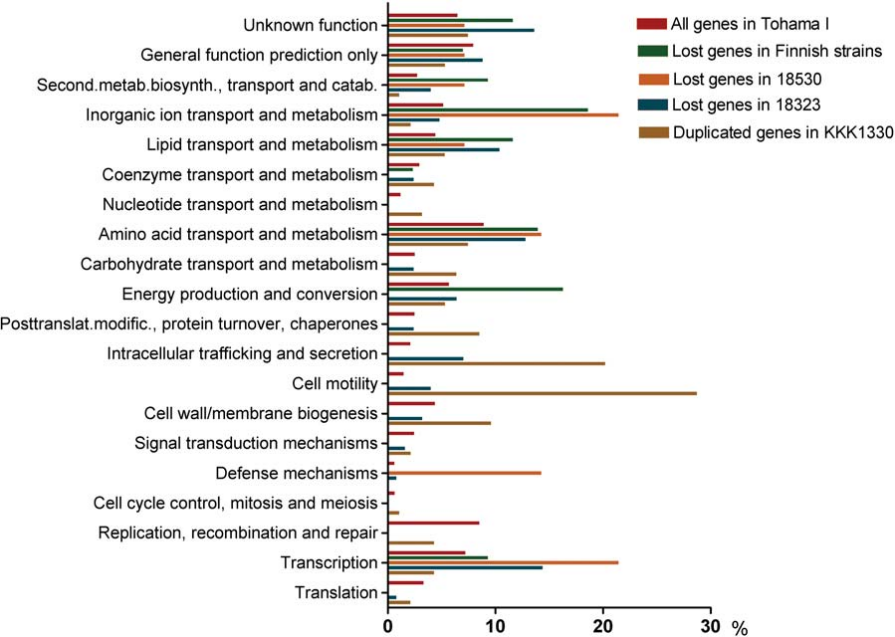
Distributions of lost and duplicated genes defined in the 20 functions based on the Clusters of Orthologous Groups. Number of the genes is shown in Table 2.

A total of 23 functional categories of genes were defined in the Tohama I strain according to the Clusters of Orthologous Groups (COG) [1]. On the basis of the COG, functions of the absent and duplicated genes were compared with that of the strain Tohama I. The number of genes which could be classified into the COG categories are shown in Table 2 and included in the comparison (Figure 3). Of the 23 functional categories of genes, 20 were included in the comparison (Figure 3), because none of the absent or duplicated genes belonged to the categories of RNA processing and modification, cell cycle control and chromatin structure and dynamics.

**Table 2:** Number of absent and duplicated genes detected in Finnish clinical strains and vaccine strain and the reference strain relative to the genome of *B. pertussis* Tohama I.

Strain (s)	No of absent genes	No of duplicated genes	No of genes defined in COG (%)	No of Pseudogenes (%)
All clinical strains	65	0	44 (67,7)	11 (16,9)
Vaccine strain 18530	22	0	12 (54,5)	5 (22,7)
Reference strain 18323	196	0	118 (60,2)	19 (9,7)
KKK 1330	7	129	92 (71,3)(1	16 (12,4)

1. No of duplicated genes defined in COG

For the clinical isolates, most notable were high frequencies of absent genes identified in the two functions: inorganic ion transport and metabolism (N = 8) and energy production and conversion (N = 7), representing one third (15/44) of total number of absent genes classified in the COG (Figure 3). The eight genes defined in the function of inorganic ion transport and metabolism included two coding for putative ferric siderophore receptors (BP1138 and BP1962), two for probable inner membrane components of binding-protein-dependent transport system (BP0914 and BP0915), putative exported protein (BP0913), putative signal transduction protein (BP1137), putative flavocytochrome (BP1961), and putative dioxygenase hydroxylase component (BP2091). The seven genes defined in the function of energy production and conversion were putative succinate-semialdehyde dehydrogenase [NADP+] (BP0919), putative exported protein (BP0920), citrate utilization protein B (BP0921), probable oxidoreductase (BP1953), putative monooxygenase (BP1954), putative ferredoxin (BP2095) and putative fatty-acyl-CoA racemase (BP2096).

There were seven absent genes identified in the function of amino acid transport and metabolism. Of the seven genes, five (BP1948-BP1951 and BP1957) were in locus 3 and two (BP2089 and BP2090) in locus 4. Four genes (BP1950, BP1951, BP2089 and BP2090) were associated with ATP binding cassette (ABC) systems which are responsible for the import and export of a wide variety of molecules across cell membranes of bacteria [23].

Of the five absent genes defined as function unknown, three (BP0923, BP0927 and BP0931) were in locus 1, one (BP1965) in locus 3 and one (BP2103) in locus 4. Four genes coded for putative exported proteins.

We have not observed any absent or duplicated genes that encoded known virulence factors of *B. pertussis*.

For the vaccine strain 18530, 12 of 22 absent genes were classified in the COG. Of the 12 genes, three belonged to the function for inorganic ion transport and metabolism (Figure 3).

For the reference strain 18323, the missing genes were found in 17 of 20 functional categories. The three categories without missing genes were replication, recombination and repair, cell cycle control, mitosis and meiosis, and nucleotide transport and metabolism (Figure 3).

For the strain KKK1330, the duplicated genes were found in the following functional categories: cell motility, intracellular trafficking and secretion, cell wall/membrane biogenesis, and posttranslational modification, protein turnover, chaperones (Figure 3). A number of duplicated genes were related to flagella.

## Discussion

A resurgence of pertussis has been observed in many countries, despite high vaccine coverage [2]–[10]. The causes for the resurgence are multiple, such as increased awareness of the disease, use of better microbiological diagnosis and different effectiveness of the vaccines used, waning vaccine-induced immunity and pathogen adaptation. Indeed, antigenic divergence has been found between *B. pertussis* vaccine strains and circulating isolates in the countries with the re-emergence of the disease [4]–[6], [8], [9], [12]–[16]. Analyses of *B. pertussis* clinical isolates using PFGE or MLVA further revealed the clonal expansion of certain strains which caused recent epidemics in the countries [6], [16]–[21].

Vaccination against pertussis has been introduced in Finland since 1952. The vaccine is produced at the National Public Health Institute, Helsinki, Finland. Strain 18530 has been used since 1962, and strain 1772 was added to the vaccine in 1976. The vaccine has not changed since then, and the vaccination coverage for four doses has been 95%. To better understand the evolution of *B. pertussis* in immunized populations, an oligonucleotide-based microarray was developed and used for comparative genomic analysis of Finnish strains isolated during a period of 50 years.

Compared with the genome of strain Tohama I, a progressive gene loss was observed among the Finnish clinical strains. Strains isolated in 1950's and 1960's had almost the same gene content as Tohama I, with only seven genes absent. A marked change with the deletion of 24 genes occurred in strains isolated in 1970's, more than 20 years after the vaccination was introduced. The gene content of the strains isolated from the following 20 years remained basically the same. The next major deletion of 18 genes was first detected in strains isolated in 1999, and the gene content of strains isolated throughout the following period remained unchanged. Our data confirmed that *B. pertussis* population is dynamic and is continuously evolving [6], [13], [21], suggesting that the bacterium uses gene loss as one strategy for its better adaptation to the highly vaccinated populations. For the future, it is of particular interest to compare our results with those obtained from strains isolated from the same period and from a non-vaccinated population.

Our results obtained from the oligonucleotide-based microarray are consistent with two recent studies of *B. pertussis* strains using cDNA microarray [24], [25]. In addition, we detected a new lost locus that contained 16 genes. However, the lost locus was only found in one among the 20 clinical strains studied.

In agreement with two recent studies, none of genes coding for known virulence factors were found to be lost in the Finnish strains [24], [25] , suggesting that virulence factors are critical for *B. pertussis* to maintain its capability in the transmission and pathogenesis among the human populations. However, changes in known virulence factors pertussis toxin, pertactin and fimbriae coincided with the loss of loci 1 and 3. Since 1977, when the locus 1 was lost, the predominant allele for the gene encoding subunit 1 of pertussis toxin had changed from *ptxA2* to *ptxA1*. Five years after the change in *ptxA*, pertactin allele shifted from *prn1* to *prn2*. Both alleles are different from the ones in the strains used for Finnish whole cell vaccine. From 1976 to 1998, about 90% of the Finnish isolates expressed Fim2. Since 1999, two nationwide epidemics occurred. In addition to the gene loss of locus 3, the common feature between the strains mainly responsible for the epidemics is the expression of Fim3.

Pseudogenes are usually formed due to frameshift mutation, mutations leading to the insertion of a stop codon or insertion of IS elements [26]. The percentage of the pseudogenes identified among the four lost loci was significantly higher than that in the whole genome, suggesting that the pseudogenes are prone to deletion during the evolution of *B. pertussis*. In addition, we have observed that the deleted regions have a slightly higher GC content compared with the whole genome (respectively, 68.7% and 67.6%).

Gene loss or genome reduction has been observed in several mammalian pathogens, and IS elements are found to play an important role in this process, in addition to causing chromosomal rearrangements and insertion mutations [27]–[30]. A recent study has shown that IS-mediated diversification in *Enterococcus faecium* strongly contributed to its adaptation to hospital environment [30].During its evolution from *B. bronchiseptica* like ancestor, *B. pertussis* has undergone significant gene loss, most likely due to IS elements [1], [31]. Three distinct IS elements were found in the genome of *B. pertussis* Tohama I strain, with the copy number of 238 for IS*481*, 6 for IS*1002* and 17 for IS*1663* [1]. Interestingly, all the four loci of lost genes identified among Finnish isolates were flanked by IS*481*. Furthermore, IS*481* was located at least on one side of all loci of lost or duplicated genes detected in this study. Taken together, the results clearly showed that IS*481* plays a critical role in the *B. pertussis* evolution.

It is of particular interest that one third of the lost genes had functions related to inorganic ion transport and metabolism and energy production and conversion. The acquisition of iron, which is an inorganic ion, is critical for pathogenic micro-organisms, because concentration of free iron is limited in the human host [32]. In order to acquire iron, bacterial pathogens produce iron-chelating compounds (siderophores). Like *B. bronchiseptica, B. pertussis* contains an operon encoding the production, export and uptake of the siderophore alcaligin [33], [34]. Iron obtained from siderophores is internalized through TonB-dependent outer-membrane ferric complex receptors. *B. bronchiseptica* has 16 genes coding for these receptors, whereas *B. pertussis* has lost 3 of the genes during its evolution from *B. bronchiseptica* like ancestor, while one of the remaining was pseudogene [1]. Two of the 12 functional genes coding for the receptors were deleted among the Finnish strains isolated from 1999. These strains were responsible for the nationwide epidemics observed in the country [6], [21]. It remains to be shown if these deletions confer a selective advantage of the epidemic-causing strains.

In this study, seven lost genes had functions in amino acid transport and metabolism, four of which encoded ATP binding cassette (ABC) transporter components and were located in two distinct loci. ABC transporters are a large group of proteins which have roles in important cellular functions such as import and export of various substances including iron chelators (siderophores) [23]. According to the Tohama I chromosome, 80 ABC transporter components have been identified [1]. Many ABC transporter components are surface associated and are involved in virulence of pathogenic bacteria [23]. It is known that the surface associated and secreted proteins usually interact with host cells, and therefore are potential immune targets. Deletion of such genes might confer a selective advantage during infection or transmission in vaccinated population. In line with this observation, we also found several lost genes which coded for putative exported proteins.

PFGE has been shown to have best discriminatory power for the study of the epidemiology of *B. pertussis*. More than 400 Finnish clinical isolates collected from 1953 have been analyzed by PFGE, and altogether about 60 PFGE profiles have been identified [6], [21]. One PFGE profile BpSR11 has emerged since 1999 and become predominant. The strains with BpSR11 were associated with the recent nationwide epidemic in the country. Interestingly, all the strains with BpSR11 analyzed in the present study had a lost locus (Locus 3) of 18 genes with the length of 22,700 base pairs.

Among the 20 clinical strains studied, only one was found to have both gene loss and duplication. The duplicated genes were associated with the functions of cell motility, intracellular trafficking and secretion, cell wall/membrane biogenesis, and posttranslational modification, protein turnoverand chaperones. Most interesting was that a number of duplicated genes were related to flagella. Flagella are usually expressed in the Bvg^−^-phase and involved in the survival of the bacteria in the environment. In *B. pertussis*, the flagellar operon was considered to be inactivated due to multiple pseudogenes and IS insertions [1]. It is of interest to study if the *B. pertussis* strain in which flagellar genes were duplicated can express the flagella and is motile.

In this study, the oligonucleotides used for microarray were designed from the genome of the Tohama I strain [1]. We were able to clearly distinguish between the presence, absence or duplication of genes among the clinical strains studied. A drawback of microarray-based comparative genomic analysis is that it cannot detect genes that are not present in the Tohama I strain. Although the acquisition of “new” genetic material does not seem to be a significant source of genetic variation in *B. pertussis* [1], [31], it is worthwhile to sequence the genome of recent *B. pertussis* isolate(s) and to show how representative the strain Tohama I, which is used for the production of a number of pertussis vaccines, is. Furthermore, sequencing of recent isolate(s) may identify genes acquired by *B. pertussis* which play a role in the persistence and resurgence of pertussis despite vaccination.

In conclusion, this microarray based on 70-mer oligonucleotides proves to be a powerful tool for comparative genomic analysis of *B. pertussis* strains. Our results showed that the progressive gene loss mediated by the homologous recombination between IS elements occurred in *B. pertussis* strains in Finland, where the vaccination against pertussis has been used for more than 50 years. The recent epidemic-causing strains showed the largest degree of gene loss compared with the vaccine strains. The high consistency in gene loss could imply the presence of antigenic determinants among lost genes and gene loss may be one strategy for *B. pertussis* to adapt to highly vaccinated populations.

## Supporting Information

Table S1 Primers used in the PCR for the confirmation of lost loci detected by microarray
(0.04 MB DOC)

Table S2 PCR results confirming the absence of four lost loci.
(0.06 MB DOC)

Dataset S1 Absent genes detected in strains 18323 and 18530 (0.01 MB XLS)
