# Supplementary material for: Comparative Genomics of Bordetella pertussis Reveals Progressive Gene Loss in Finnish Strains
Source: PLoS One. 2007 Sep 19;2(9):e904. doi: 10.1371/journal.pone.0000904 (PMC1975675; doi:10.1371/journal.pone.0000904)
Supplement: Table S1 — Primers used in the PCR for the confirmation of lost loci detected by microarray (0.04 MB DOC) [file pone.0000904.s001.doc]

Table S1. Primers used in the PCR for the confirmation of lost loci detected by microarray

| Locus | Primer sequence | Product length (bp) | Target gene |
| --- | --- | --- | --- |
| 1 | 5’-CAC GGT GTT GAA GCT GAA GA-3’ | 3,493 | BP0909 |
|  | 5’-GGC CAC GAT GTA GAA CAC CT-3’ |  | BP0913 |
|  | 5’-CCG TAG ATG GTG CCT ATC GT-3’ | 2,879 | BP0933 |
|  | 5’-AAG TGG TCT GCC AGC GTA GT-3’ |  | BP0936 |
| 2 | 5’-GGA TAG CCC GAC TCC TTC AC-3’ | 4,127 | BP1131 |
|  | 5’-TAT TGC TGC ACC GAA CTG AG-3’ |  | BP1136 |
|  | 5’-CTT CTA CCG CTT CCA TTT CG-3’ | 236 | BP1140 |
|  | 5’-TGC GCT GAC TGT ATT TCC TG-3’ |  | BP1140 |
| 3 | 5’-GTG GCG ATC TTT CAG CAG AC-3’ | 3,266 | BP1946 |
|  | 5’-TAC ACG CCC AGC ATG TAG AG-3’ |  | BP1949 |
|  | 5’-ACT TCT TCG CCC TCA TGC T-3’ | 4,024 | BP1965 |
|  | 5’-GCC AGA TCG AGT TCG AGG TA-3’ |  | BP1969 |
| 4 | 5’-CGC CTG AAG GGC TAC TAC CT-3’ | 340 | BP2088 |
|  | 5’-ATA CGA CAA ACG CCT TGA GC-3’ |  | BP2088 |
|  | 5’-ACG ACA AAC AAG GGG AAG TG-3’ | 3,766 | BP2103 |
|  | 5’-TTG AAG ATC GGT CCC AAC TC-3’ |  | BP2107 |

Target gene indicates the gene from which the primer is derived.
