# Supplementary material for: Comparative Genomics of Bordetella pertussis Reveals Progressive Gene Loss in Finnish Strains
Source: PLoS One. 2007 Sep 19;2(9):e904. doi: 10.1371/journal.pone.0000904 (PMC1975675; doi:10.1371/journal.pone.0000904)
Supplement: Table S2 — PCR results confirming the absence of four lost loci. (0.06 MB DOC) [file pone.0000904.s002.doc]

Table S2. PCR results confirming the absence of four lost loci.

| Strain | Lost loci | | | | | | | |
| --- | --- | --- | --- | --- | --- | --- | --- | --- |
|  | BP0910A-BP0934 | | BP1135-BP1141 | | BP1948-BP1966 | | BP2088-BP2103 | |
|  | 3' | 5' | 3' | 5' | 3' | 5' | 3' | 5' |
| Tohama I | + | + | + | + | + | + | + | + |
| 1772 | - | - | - | - | + | + | + | + |
| 18530 | + | + | + | + | + | -/+ | + | + |
| KKK22 | + | + | - | - | + | + | + | + |
| KKK1277 | + | + | - | - | + | + | + | + |
| KKK1330 | + | + | - | - | NA. | + | + | + |
| 1977/3 | - | - | - | - | + | + | + | + |
| 1977/7 | - | - | - | - | + | + | + | + |
| PRCB2/H | - | - | - | - | + | + | + | + |
| PRCB20/S | - | - | - | - | + | + | + | + |
| PRCB2 | - | - | - | - | + | + | + | + |
| PRCB13 | - | - | - | - | + | + | + | + |
| PRCB41 | - | - | - | - | + | + | + | + |
| PRCB179 | - | - | - | - | + | + | + | + |
| PRCB223 | - | - | - | - | + | + | - | - |
| PRCB272 | - | - | - | - | - | - | + | + |
| PRCB291 | - | - | - | - | - | - | + | + |
| PRCB305 | - | - | - | - | - | - | + | + |
| PRCB374 | - | - | - | - | - | - | + | + |
| PRCB382 | - | - | - | - | - | - | + | + |
| PRCB406 | - | - | - | - | - | - | + | + |
| PRCB461 | - | - | - | - | - | - | + | + |
| PRCB474 | - | - | - | - | - | - | + | + |

## + or - indicates strain from which specific PCR product was or was not obtained. NA, not analyzed.
